# Trace Elements and Heavy Metal Contents in West Algerian Natural Honey

**DOI:** 10.1155/2022/7890856

**Published:** 2022-12-30

**Authors:** Dalila Bereksi-Reguig, Salim Bouchentouf, Hocine Allali, Agnieszka Adamczuk, Grażyna Kowalska, Radosław Kowalski

**Affiliations:** ^1^Department of Chemistry, Faculty of Sciences, Abou Bekr Belkaïd University, P.O. Box 119, Tlemcen 13000, Algeria; ^2^Doctor Tahar Moulay University of Saida Algeria, BP 138 Cité EN-NASR, Saïda 20000, Algeria; ^3^Laboratory of Natural and Bioactive Substances (LASNABIO), Department of Chemistry, Faculty of Sciences, Abou Bekr Belkaïd University, P.O. Box 119, Tlemcen 13000, Algeria; ^4^Institute of Agrophysics, Polish Academy of Sciences, Doświadczalna 4, Lublin 20-290, Poland; ^5^Department of Tourism and Recreation, University of Life Sciences in Lublin, 15 Akademicka Street, Lublin 20-950, Poland; ^6^Department of Analysis and Food Quality Assessment, University of Life Sciences in Lublin, 8 Skromna Str., Lublin 20-704, Poland

## Abstract

Analysis of trace elements and heavy metals in honey is essential for honey quality and safety and also monitoring environmental pollution. This study aimed to evaluate the composition of thirty-seven honey samples of different botanical origins (14 multifloral and 23 unifloral) obtained from beekeepers located in the west region of Algeria. Inductively coupled plasma-mass spectrometry (ICP-MS) and atomic absorption spectroscopy (AAS) methods were used to determine the levels of 19 elements in honey (K, Na, Ca, Mg, Mn, Cu, Fe, Zn, V, Cr, Co, As, Ru, Rh, Cd, W, Pt, Au, and Pb). Ru, Rh, Pt and, Au were not detected in any of the tested honey samples. The most abundant minerals were K, Ca, Na, and Mg ranging within 153.00–989.00 mg/kg, 33.10–502.00 mg/kg, 13.30–281.00 mg/kg, and 20.80–162.00 mg/kg, respectively. Fe, Mn, Zn, and Cu were the most abundant heavy metals while Pb, V, Cr, W, Co, and Cd were the lowest ones (<1 mg/kg) in the honey samples surveyed. Several honey types, lavender, rosemary, mild white mustard, thyme, milk thistle, carob tree, orange tree, *Euphorbia*, *Eucalyptus*, camphor, jujube tree, sage, and harmal, were studied, and the statistical analysis was carried out using principal component analysis (PCA) and hierarchical cluster analysis (HCA) techniques to evaluate the data. The results showed that the analyses of mineral content were sufficient to determine the floral origin and their variability may be related to geochemical and geographical differences. On other hand, all elements detected were at levels below safe thresholds.

## 1. Introduction

Bees are one of the most useful insects for humans: they pollinate our crops and produce honey [1, 2]. Nowadays, they play a new role in indicating pollution and the quality of the environment, through produced honey [3, 4]. The bee forages for several kilometers in search of nectar; on its way, its body accumulates particles of metals and pollutants present in the environment which it transports to the hive [5].

Contaminants can be anthropogenic or natural and may pollute the soil with different fractions in many ways [6]. Pollutants have been shown to be absorbed by plants growing in contaminated soil and have higher levels in their tissue compared to other plants grown in control soils [7, 8]. There is a close relationship between beehive products, notably honey, and plants, which means that honey inherits various characteristics and biological properties of plants with respect to their respective botanical sources and growing surface [9]. Due to this relationship, undesirable compounds or residues can be found in honey if plants or soil have been exposed to these substances [10]. Among the most hazardous contaminants for human health and the environment which can be found in honey, we can cite heavy metals [11, 12]. They are found naturally in the Earth's crust, but their presence in the environment has increased due to high anthropogenic activity [13]. For several decades, it has been shown that exposure to metals implies serious damage [14]. Their bioaccumulation and amplification in living systems lead to undeniable effects and diseases [15, 16]. Qualitative and quantitative analyses of heavy metals in honey, in addition to the classical approach, would allow a reliable and reproducible system to establish the botanical origin [17] and geographic traceability [18]. The environmental aspect of a region has also been assessed using minerals and heavy metals present in honey [19]. Several studies have been conducted to establish the relationship between pollution in a region and the presence of contaminants in honey [20–22]. The main elements found in different kinds of honey throughout the world, considered as a profiling parameter, can be divided into two groups according to the literature: major elements or gold macro-elements (Na, K, Ca, Mg, P, S, and Cl) and trace elements including heavy metals (Al, Cu, Pb, Zn, Mn, Cd, Tl, Co, Ni, Rb, Ba, Be, Bi, U, V, Fe, Pt, Pd, Te, Hf, Mo, Sn, Sb, La, I, Sm, Tb, Dy, Sd, Th, Pr, Nd, Tm, Yb, Lu, Gd, Ho, Er, Ce, Cr, As, B, Br, Cd, Hg, Se, and Sr) [20, 22–28]. The latter can affect the nervous system, kidney, liver, and respiratory functions. Some metals, such as cadmium, arsenic, nickel, and chromium, are carcinogenic [29]. Exposure to heavy metals is implicated in more severe pathologies like multiple sclerosis and neurodegenerative diseases (Alzheimer's and Parkinson's disease) [29–31]; they could even play a role in triggering psychological and neurological disorders such as autism [32]. It is also important to mention that the presence of certain minerals in honey is essential for the evaluation of its nutritional and curative quality [33]. Besides minerals and trace elements, honey is composed of various carbohydrates, polysaccharides, oligosaccharides, flavonoids, vitamins, minerals, waxes, aroma compounds, pollen grains, pigments, and enzymes which also contribute to its profiling and authentication [34]. In Algeria, honey is considered as a precious product because of its high nutritional value as well as its therapeutic virtues widely popular traditionally [35, 36]. The relatively mild climate in Algeria and abundant varied flora allow the production of a variety of honey [37]. According to the Algerian Center for Quality Control and Packaging (CACQE), national honey production was estimated in 2011 at 33,000 quintals and in the same year 150,000 tonnes of honey, mainly from China, India, and Saudi Arabia, were imported (CACQE, https://www.cacqe.org/). The growing interest in honey, which is considered as a noble substance, requires vigilance to its authenticity and originality to preserve national production, avoiding every sort of fraud. Based on multiple searches done on the PubMed database using different keywords, it is noteworthy that the number of studies on the physicochemical properties of Algerian honey, particularly in the west region, is very scarce. The present study aims to identify major and trace elements contained in different samples of honey collected in west Algeria. Besides physicochemical methods, we used principal component analysis (PCA) and hierarchical clusters analysis (HCA) techniques to differentiate Algerian honey, from different botanical origins, according to their mineral content and the choice of elements with a higher discriminating power.

## 2. Materials and Methods

### 2.1. Honey Samples

**Figure 1:**
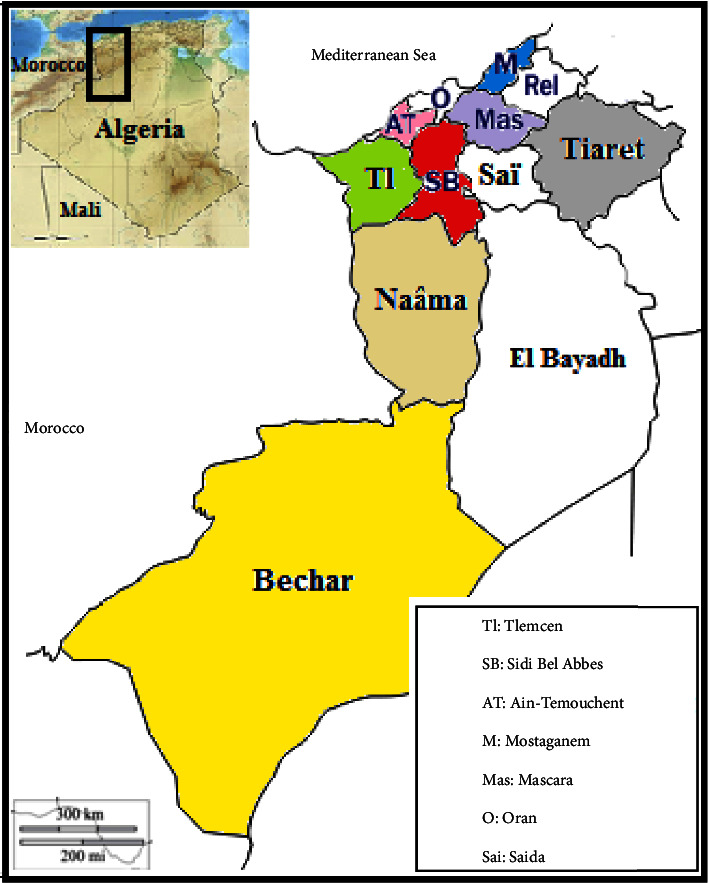
Locations of the sampling points in west Algeria.

A set of thirty-seven *Apis mellifera intermissa* honey samples (500 g each), namely, S1–S37, was collected in 2017 and 2018 from eight geographical provinces (Tlemcen, Ain Temouchent, Sidi Bel Abbes, Mostaganem, Mascara, Tiaret, Naâma, and Bechar) in west Algeria (Figure 1).  Table 1 shows the botanical and geographical origins of the honey samples studied. Samples were taken directly from beekeepers with a guaranteed origin, stored in airtight plastic containers, and then kept in a refrigerator at 4°C until ICP-MS and AAS analysis processes.

### 2.2. Reagents and Solution

High-purity deionized water acquired by passing distilled water through a water purification system (demineralizer HLP 20, Hydrolab, Poland) was used to prepare the solutions. Certified single-element standard solutions (1000 mg/L) used to prepare the calibration curve were of the highest purity grade (99.999%) and were supplied by Ultra Scientific (North Kingstown, RI, USA). Other reagents were of analytical grade unless otherwise stated. Honey samples were digested with Suprapur® grade nitric acid (HNO_3_ 65% m/m, Merck, Germany). A recovery test was performed using a single-element solution and two reference materials, namely, Standard Reference Materials (SRMs) Tomato Leaves and Pine Needles (*Pinus taeda*) (SRM 1573a and SRM 1575a, resp., National Institute of Standards and Technology (NIST), Gaithersburg, USA).

### 2.3. Instrumentation

Elemental analysis was carried out by using 820-MS inductively coupled plasma quadrupole mass spectrometer (ICP-MS; Varian, Mulgrave, Australia) equipped with an SPS3 autosampler (Varian, Australia) and a MicroMist nebulizer type (Varian). A MARS Express microwave mineralizer system (CEM, Matthews NC, USA) of Teflon reaction vessels was used in the digestion procedures. The reaction vessels were cleaned using 10 mL of concentrated nitric acid before each digestion. The multielemental determination was also determined by the atomic absorption spectroscopy (AAS) method using a spectrometer SpectrAA 280 FS with autosampler SPS3 (Varian, Australia), which was equipped with a deuterium lamp, hollow cathode lamp for each element, and an air-acetylene burner.

Appropriate results in terms of accuracy and sensitivity, low cost, and quickness make AAS a suitable procedure for determining the concentrations of alkaline and earth alkaline elements in the honey samples investigated. Tables 2 and 3 show the instrumental parameters for ICP-MS.

### 2.4. Analytical Determination

Trace element and heavy metal determination was performed by the inductively coupled plasma-mass spectrometer (ICP-MS) and atomic absorption spectroscopy (AAS) using the operating conditions and emission wavelength lines listed in Tables 2 and 3. Glass and plastic material was cleaned and kept in 10% (v/v) nitric acid solution for at least two days. The material was then rinsed three times with high-purity deionized water before being used. Approximately 0.5 g of each honey sample was digested with 10 mL of 65% HNO_3_ (v/v) in Teflon vessels. The sealed vessels were put into the microwave mineralizer MARS Express (CEM, USA). A blank digest was carried out in the same way. The microwave mineralization was performed stepwise at 400 W and 363 K, at 800 W and 393 K, and at 1600 W and 483 K. The cooled digestion solution was then diluted to 50 mL using high-purity deionized water. This solution was finally used for the determination of V, Cr, Co, As, Ru, Rh, Cd, W, Pt, Au, and Pb of the honey samples, performed with an inductively coupled plasma-mass spectrometer equipped with a concentric nebulizer, a quartz torch with quartz injector tube, and cyclonic spray chamber (Table 2). The concentration of K, Na, Ca, Mg, Mn, Cu, Fe, and Zn ions was determined by the AAS method using a spectrometer SpectrAA 280 FS with autosampler SPS3 (Table 3). To avoid sample ionization during potassium analysis, Schinkel buffer solution (mixture contents: 10 g/L cesium chloride and 100 g/L lanthanum chloride) was used. Each sample was measured in triplicate by AAS and ICP-MS detection.

### 2.5. Quality Control

The analytical quality was controlled by means of certified reference materials: NIST-1573a Tomato Leaves and NIST-1575a Pine Needles. The certified reference materials were prepared according to the instructions of the manufacturer. Honey samples and quality control samples (blind samples and certified reference materials) were determined in triplicate, and the average was given as the final result.  Table 4 presents the validation parameters obtained during analysis. For Au, Pt, Rh, Ru, and W, the recovery parameter was obtained from fortification by using certified single-element standard solutions.

### 2.6. Statistical Method

Principal component analysis (PCA) and hierarchical cluster analysis (HCA) were performed with the XLSTAT 2014.5.03 software for Microsoft Excel (Addinsoft, Bordeaux, France) to classify and discriminate the honey samples.

## 3. Results and Discussion

### 3.1. Results of Minerals in Honey Samples

The concentrations of trace element and heavy metals found in the honey samples collected from thirty-seven locations situated in west Algeria are given in Tables 5 and 6. Validation parameters of the analytical procedure such as: limit of detection (LOD), limit of quantification (LOQ), precision, accuracy, and uncertainty budget are listed in Table 4. A detailed validation of the analytical procedure was performed including the performance parameters limit of detection (LOD) and limit of quantification (LOQ); precision, accuracy, and an uncertainty budget (Table 4).

Precision and percentage recovery of the analytical procedure were determined by using randomly selected honey samples individually spiked with known spiked concentrations of the trace elements and heavy metals studied (K, Na, Ca, Mg, Mn, Cu, Fe, Zn, V, Cr, Co, As, Ru, Rh, Cd, W, Pt, Au, and Pb) and used as positive controls. The mean percentage recoveries of the determined trace elements and heavy metals ranged between 74.51% (Co) and 117.32% (Fe) (Table 4), which point out good accuracy, precision, and validity of the method employed. The results of mineral content in honey were found relatively low and varied over a range within 0.04–0.16% of the total composition, which is in agreement with the composition of nectar honey [22, 38]. A total of 19 trace elements and heavy metals were determined. It should be noted that the concentrations of 19 elements were variable depending on the floral origin of honey [19]. Among them, the most abundant elements were K, Ca, Na, and Mg with average levels (mean ± SD) of 532.46 ± 212.69 mg/kg, 113.92 ± 95.38 mg/kg, 83.40 ± 64.85 mg/kg, and 100.83 ± 33.88 mg/kg, respectively. The west Algerian honey samples in the present investigation showed a wide range of K contents (153.00–989.00 mg/kg) accounting for 80.08% of the total minerals present in honey from Bouzedjar (S21). Following our data, similar amounts of K in honey samples were previously reported from the Azilal and Beni Mellal provinces in Moroccan (256–1023 mg/kg) [39] and Tunisian (172.48–976.75 mg/kg) honey [40]. Overall, the concentrations of K in west Algerian honey were higher than those reported from Turkey (1.18–268 mg/kg) [20], the West Bank in Palestine (42.80–585.00 mg/kg) [41, 42], and Jableh and Tartous provinces in the western part of Syria (38.2–174 mg/kg) [43], but the levels were lower than those reported for Portugal (117.55–2590.60 mg/kg) [44, 45], Italy (237–6520 mg/kg) [23, 25], Libya (1120.1–1980.6 mg/kg) [46], and Spain (1615–3770 mg/kg) [47–52]. *Eucalyptus* honey showed the second highest concentration (987.00 mg/kg, S27) of K among the honey samples investigated while carob tree honey contained the lowest amount (153.00 mg/kg, S12). High concentrations of K were also present in the mild white mustard (946.00 mg/kg, S6), lavender (808.00 mg/kg, S1), and milk thistle (802.00 mg/kg, S23) honey when compared to the other honey samples studied. The concentration of K in the present study is also much higher than the three *Citrus* spp. (Citrus) honey samples from Syria (38.2–174 mg/kg) [43]. This discrepancy may be due to the geographical variation in the sources of honey [52]. A high mean value (113.92 ± 95.38 mg/kg) of Ca was observed among the investigated west Algerian honey samples with the concentrations ranging within (33.10–502.00 mg/kg) which were higher than those of honey samples reported for Morocco (19.71–200.1 mg/kg) [39], Tunisia (113.85–221.07 mg/kg) [40], Portugal (6.24–134.35 mg/kg) [44, 45], Spain (11.69–218.5 mg/kg) [47–49, 52], France (8.90–130.90 mg/kg) [53, 54], Italy (<43–283 mg/kg) [23, 25], Turkey (<0.001–4.5 mg/kg) [20], Palestine (44.50–150.70 mg/kg) [41, 42], Syria (43.3–118 mg/kg) [43], Greece (15.22–65.93 mg/kg), Cyprus (23.66–143.47 mg/kg), and Egypt (44.79–112.10 mg/kg) [26] (Tables 7 and 8). Besides, honey samples collected from different locations in the west of Libya show the highest concentrations with a range within (923.92–1117.5 mg/kg) [46] of all the Mediterranean regions considered. It would be interesting, due to the presence of the high amount of this mineral, to propose it in a strategy for the prevention of osteoporosis. Sage honey showed the highest concentration (502.00 mg/kg, S34) of Ca, followed by multifloral honey (377.00 mg/kg, S14), *Eucalyptus* honey (287.00 mg/kg, S25), and milk thistle honey (215.00 mg/kg, S23). Interestingly, there are some similarities between the Ca contents of some types of west Algerian honey and those coming from neighboring countries. For instance, the Ca concentration of *Eucalyptus* honey is similar to that of carob honey from Rabat province in Morocco (286.01 ± 5.79 mg/kg) [55], while milk thistle honey has a similar Ca concentration when compared to the Tunisian mint honey (221.07 ± 5.16 mg/kg) [40]. The concentrations of Na in the west Algerian honey samples were higher than those reported for honey from Portugal (0.36–95.13 mg/kg) [45], Spain (11–84 mg/kg) [51], Italy (4.8–176 mg/kg) [23, 25], and Turkey (0.48–13.1 mg/kg) [20]. *Euphorbia* and *Eucalyptus* honey contained the highest concentrations of Na (281.00 mg/kg, S22; 275.00 mg/kg, S25, resp.) among the honey types while thyme, lavender, and milk thistle honey had the lowest amounts (13.30 mg/kg, S7; 21.60 mg/kg, S1; 22.10 mg/kg, S8, resp.). Other types of west Algerian honey such as orange tree, jujube tree, and mild white mustard honey samples are also rich in Na (179.00 mg/kg, S28; 165.00 mg/kg, S32; 146.00 mg/kg, S6, resp.) when compared to the other honey samples investigated. On the other hand, it should be noted that the higher concentrations of K and Na were exhibited by mild white mustard honey collected from Aïn Fezza (S6) located in Tlemcen province (Table 1). The high content of both K and Na in mild white mustard honey makes it less dangerous when consumed by hypertensive patients. Magnesium was the fourth most abundant element in the present study, with contents ranging from 20.80 to 162.00 mg/kg. These concentrations were similar to those of Italian honey (22–159 mg/kg) as reported by Pisani et al. [23] and were higher than those coming from most countries of the Mediterranean region (Table 7) except honey from Portugal (2.77–234.63 mg/kg) [45] and Spain (30.00–402.00 mg/kg) [51]. Milk thistle honey contained the highest concentration (162.00 mg/kg, S8) of Mg among the honey samples investigated, while multifloral honey samples S24 and S36 (20.80 mg/kg and 42.50 mg/kg, resp.) contained the lowest. High concentrations of Mg were also exhibited by thyme (159.00 mg/kg, S7), mild white mustard (149.00 mg/kg, S6), and lavender (142 mg/kg, S1) honey types, which are similar to the honey from Spain (159 mg/kg) [23] and France (145 mg/kg) [54]. In the case of Fe, its concentrations in the west Algerian honey ranged from 8.48 to 59.60 mg/kg. Honey samples from the Mediterranean countries reported lower Fe contents (Table 7). It is to highlight that the highest Fe levels were found in lavender honey (59.60 mg/kg, S1), while multifloral (8.48 mg/kg, S24), *Eucalyptus* (11.30 mg/kg, S25), and milk thistle (11.70 mg/kg, S8) honey had the lowest contents. Our values were well below the provisional tolerable weekly intake (PTWI) by body weight (5.6 mg/kg b.w.) recommended by the Joint FAO/WHO Expert Committee on Food Additives (JECFA) [56]. The concentration of Mn in the investigated west Algerian honey was between 1.36 and 13.90 mg/kg. Among the honey samples investigated, mild white mustard honey is the richest in Mn (13.90 mg/kg, S6). High concentrations of Mn were also shown by the milk thistle and lavender honey (13.40 mg/kg, S18; 13.30 mg/kg, S1, resp.) as well as thyme (12.70 mg/kg, S7) honey. The levels of Mn in honey samples surveyed in this study were higher than those reported for most honey samples coming from the Mediterranean region (Table 7). In the present study, the Zn concentrations ranged from 0.22 to 13.90 mg/kg (mean ± SD 3.61 ± 2.35 mg/kg). The highest Zn level was 13.90 mg/kg in carob tree honey (S10), while the lowest one was 0.22 mg/kg in multifloral honey (S5). The levels of Zn in the west Algerian honey are lower than those from Palestine (0.13–25.20 mg/kg and 1.00–19.90 mg/kg) [41, 42], respectively, but are higher than those from Morocco (≤0.1–0.69 mg/kg) [39], Tunisia (0.42–2.06 mg/kg) [40], Portugal (0.03–3.29 mg/kg) [45], Spain (2.34–3.47 mg/kg) [50], France (nd–1.4 mg/kg) [54], Italy (0.72–3.66 mg/kg) [23], Turkey (<1–237 *μ*g/kg) [20], Greece (0.97–9.30 mg/kg), Cyprus (0.86–6.94 mg/kg), Egypt (0.55–1.68 mg/kg) [26], and Syria (0.206–2.76 mg/kg) [43]. In the case of the concentrations of Zn, up 91% of our honey samples are in the maximum tolerable weekly intake range (2.1–7 mg/kg b.w.) [56]. The concentrations of Cu in the west Algerian honey ranged from 1.66 to 9.62 mg/kg (mean ± SD 4.90 ± 1.64 mg/kg). The highest concentrations of Cu were in multifloral, jujube tree, and carob tree honey (9.62 mg/kg, S4; 8.97 mg/kg, S33; and 7.86 mg/kg, S10, resp.), but Cu was not detected in more than a quarter of the investigated honey. Unfortunately, the levels in approximately 54% of the investigated honey samples are above the PTWI (3.5 mg/kg b.w.) for Cu established by Joint FAO/WHO [56]. Generally, Cu is transferred and accumulated in food under the influence of the environment and also due to human contributions (fertilizers and pesticides) [57]. Copper levels in west Algerian honey are slightly higher than those from Morocco (≤0.1 mg/kg) [39], Tunisia (0.12–0.34 mg/kg) [40], Portugal (0.00–5.35 mg/kg) [45], France (0.06–1.71 mg/kg) [53], and other Mediterranean countries (Table 7). In summary, *Eucalyptus* honey contained the highest K and Na values. Sage honey is rich in Ca while milk thistle honey is rich in Mg. Lavender honey contained high Fe levels while the carob tree is the richest in Zn. Our study shows also that the mild white mustard honey contained highest level of Mn, whereas jujube tree and carob tree honey comprised Cu in high concentrations. Generally, the west Algerian honey samples are rich in minerals. The latter contains a significant proportion of microelements and therefore are valuable food products. These latter are a significant fraction of micronutrients making honey valuable food products. Some of these elements are vital, and others are merely desirable or beneficial. Indeed, they have significant roles in the activation of certain enzymes; these include Mg, Fe, Mn, Zn, and Cu, which play an important role in the metabolic transformations in the human body, while K, Ca, and Na are essential in building strong bones and teeth, muscle contractions, nerve signals, regulating heartbeat, and fluid balance within cells [58]. Their deficiencies play critical roles in many disorders such as hypertension and osteoporosis [59]. Some honey samples studied are important sources of micronutrients useful to the proper functioning of the human body, and therefore their consumption is highly recommended, especially in case of deficiency. To verify the quality of the west Algerian honey, in addition to Mn, Fe, Zn, and Cu, it is very important to assess and monitor the concentrations of other heavy metals and metalloids, which are potentially toxic. They include arsenic, lead, chromium, cadmium, vanadium, tungsten, cobalt, ruthenium, rhodium, platinum, and gold. In the present study, the relative concentrations of these elements in the honey samples decreased in the following order: Pb > V > Cr > W > As > Co > Cd > Ru, Rh, Pt, and Au. All of them were detected at levels <1 mg/kg. The resulting data, summarized in Table 5, were consistent with the ranges indicated for honey from other studies [20, 26, 50, 53, 60]. Lead is considered a strict contaminant and is toxic to living organisms, even at very low concentrations. Its content in honey is examined in several studies. The range values of Pb content in honey samples from the west Algerian region were 0.54–132.73 *μ*g/kg. Pb was found at high concentrations in the multifloral honey samples (132.73 *μ*g/kg, S17; 91.36 *μ*g/kg, S30; and 89.44 *μ*g/kg, S21) and was absent in milk thistle (S8). Furthermore, the observed concentrations of Pb were higher than those measured in Morocco (≤0.1 mg/kg) [38], Tunisia (0.01–0.05 mg/kg) [40], Greece (<0.08 mg/kg), Cyprus (<0.08 mg/kg), Egypt (<0.08 mg/kg) [26], Spain (46.32–31.50 *μ*g/kg) [50], and Syria (<0.082 mg/kg) [43]. However, the concentrations of Pb in the examined honeys were lower than in the following honeys: Palestinian (0.51-0.94 mg/kg) [42], French (3-101 mg/kg) [54], and Italian (28-304 mg/kg) [23], and (9-209 mg/kg) [25]. However, concentrations found were lower than those found in Palestinian (0.51–0.94 mg/kg) [42], French (3–101 mg/kg) [54], and Italian (28–304 mg/kg, [23]; 9–209 mg/kg, [25] honey. In this study, the mean level of Pb in honey samples is 24.73 *μ*g/kg, which is still within the PTWI of Pb for adults (25 *μ*/kg b.w.) [56]. The extremely high Pb levels found in Italian honey depicted contamination caused by external sources or by incorrect procedures during honey processing as reported by Pisani et al. [23]. Vanadium is a natural component of the Earth's crust, which is widespread in nature. One of the main sources of environmental pollution by V comes from the combustion of fossil fuels [61]. The mean value of V content in honey samples from the thirty-seven locations in west Algeria was 13.52 *μ*g/kg. Among the honey samples surveyed, camphor and thyme honey samples are the richest in V (106.51 *μ*g/kg, S26; 79.73 *μ*g/kg, S11, resp.), whereas rosemary honey (0.81 *μ*g/kg, S29) was the poorest one. In this study, the levels of V were lower than those reported by Conti et al. [25] (Table 7). There is currently no JECFA assigned reference health standard for vanadium. Chromium is abundant in the environment in a trivalent or hexavalent state. It is found in the trivalent state, at low concentrations, in a wide range of foods [62]. Its content in foods can be greatly affected by anthropic and geochemical factors. The mean and the range values of the Cr content of honey samples investigated were 46.65 and 27.36–87.37 *μ*g/kg. Levels observed were lower than those reported in Tunisia (0.02–0.32 mg/kg) [40], France (0.08–0.36 mg/kg) [53], Palestine (0.00–0.51 mg/kg) [41], and Spain (0.049–4.480 mg/kg) [60]. On the other hand, very low Cr levels were found in *Eucalyptus*, thyme, and rosemary (27.36 *μ*g/kg, S27; 27.63 *μ*g/kg, S7; and 27.81 *μ*g/kg, S29, resp.) honey samples, whereas carob tree contained the highest concentration (87.37 *μ*g/kg, S10). Chromium has low toxicity in foods in part due to its low bioavailability. Currently, there is no formal recommended dietary allowance for Cr [63]. Tungsten is found naturally on Earth almost exclusively in the combined state with other elements in ores as wolframite and scheelite. Because it is a rare metal and its compounds are generally inert, the effects of W on the environment are limited [64]. Few honey samples from the west Algerian region contained quantifiable concentrations of W. The highest W concentration was found in harmal honey (24.30 *μ*g/kg, S35). Besides, W concentrations of approximately 90% of honey samples were below LOQ. W was not investigated in others studies around the Mediterranean region. Therefore, it is not possible to comment on any differences in the level of W in honey. Arsenic is a ubiquitous element with metalloid properties. It occurs naturally in organic and inorganic forms. This is the most toxic element and is considered carcinogenic to humans [65]. The major use of As compounds is in agriculture and forestry as pesticides and herbicides [66]. The levels of As ranged from 1.61 to 21.56 *μ*g/kg with a mean concentration of 5.86 *μ*g/kg (Table 5). The highest levels of As were found in the multifloral and milk thistle honey (21.56 *μ*g/kg, S4 and 18.20 *μ*g/kg, S8, resp.) while its concentration was lower than LOQ in lavender honey (S1). Also, low concentrations were found in orange tree and thyme honey (1.61 *μ*g/kg, S16 and 1.75 *μ*g/kg, S7, resp.). Our values for As were similar to the Libyan values (0.006–0.018 mg/kg) [46] and were below those from Morocco (0.00–0.045 mg/kg) [67], Greece, Cyprus, and Egypt (<0.08 mg/kg) [26]. Very high As contents have been reported in some honey samples from France (nd–8.0 mg/kg) [54] and Italy (<25 mg/kg; 2.8–11.1 mg/kg, resp.) [23, 25], which may be from environmental contamination. Up 94% of our values were below the PTWI of inorganic As (15 *μ*g/kg b.w.) established by JECFA [56]. Low concentrations of Co (0.36–21.19 *μ*g/kg) were observed in the investigated west Algerian honey samples, which were lower than those previously reported for Moroccan (0.00–1.435 mg/kg) [67], French (0.10–0.23 mg/kg) [53], Spanish (0.015–0.720 mg/kg) [60], and Italian (1.0–17 mg/kg and 2.9–30.2 mg/kg, resp.) [23, 25] honey. Besides, the levels of Co were similar to those published for honey coming from Greece, Cyprus, and Egypt (<0.03 mg/kg) reported by Karabagias et al. [26]. Highest concentrations of Co were found in harmal honey (21.19 *μ*g/kg, S35), whereas the lowest concentrations were in multifloral and orange tree honey (0.36 *μ*g/kg, S17; 1.02 *μ*g/kg, S16, resp.). Overall, our data indicate that there are low levels of Co contamination in the west Algerian honey samples. There is currently no upper level of intake established by WHO and JECFA for cobalt. Cadmium is a metallic element that occurs naturally at low concentrations in the environment. It has been added to the environment through anthropogenic activities such as Cd metal production in industrial processes or by the use of phosphate fertilizers in agricultural soils [68]. The level of Cd in the west Algerian honey samples ranged from 0.24 to 8.14 *μ*g/kg, which was lower than that reported for the honey from most of the Mediterranean countries. The levels of Cd of half of them are, however, higher than those from Turkey (<1 *μ*g/kg) as reported by Altun et al. [20] (Table 8). Moreover, Cd was present at a high concentration in thyme honey (8.14 *μ*g/kg, S7) and below the limit of quantification in multifloral honey (S17). In 2005, the Joint FAO/WHO established a PTWI for Cd of 7 µg/kg b.w. [66]. It is worth noting that the average Cd content (1.23 μg/kg) for the examined honeys was lower than the PTWI value. [66]. Ruthenium, rhodium, and platinum are part of the platinum-group metals belonging to the group VIII transition metals. They are generally grouped with gold and silver as precious metal commodities. Each of the metals occurs naturally in its native form, and in economically exploitable deposits, the elements occur overwhelmingly as mineral species. The uses of these metals are distributed among the chemical, electrical, jewellery, and glass industries. In order to find out if these metals have found their way into the west Algerian honey, their levels were monitored in all the samples considered. In this study, the results show that Ru, Rh, Pt, and Au were lower than the limit of quantification in any of the tested honey samples. In summary, the levels of heavy metals and metalloids were, generally, low and comparable with the levels in honey from uncontaminated regions [20, 39, 40], indicating that the west Algerian honey is of good quality.

### 3.2. Multivariate Statistical Methods

**Figure 2:**
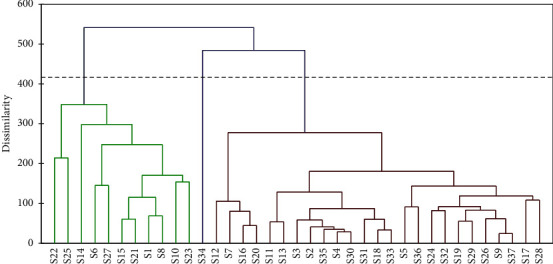
Dendrogram of analyzed honey samples.

The principal component analysis (PCA) and hierarchical cluster analysis (HCA) of the 37 honey samples were performed by XLSTAT 2014.5.03 software for the trace elements and heavy metals shown in Tables 5 and 6 except Ru, Rh, Pt, and Au, which were not detected excluded. The variance estimate results (eigenvalues) obtained are presented in Table 9. PCA is a very powerful pattern recognition technique that describes the variance of a large dataset of intercorrelated variables with a lesser set of independent variables [27]. Analyzing the data in Table 9, it can be observed that PCA account contains together for 60.89% of the total variance of the dataset. The first component eigenvalues 1.937 and cumulative percentage is 30.38%, second component eigenvalues 1.607, cumulative 54.33%, third component eigenvalues 1.562, cumulative 54.79% and fourth component eigenvalues 1.209, cumulative 60.89% of the total variance. From Table 9 it can be observed that PCA account contains together for 60.89% of the total variance of the dataset. The first component eigenvalues 1.937 and cumulative % is 30.38, second component eigenvalues 1.607, cumulative 54.33%, third component eigenvalues 1.562, cumulative 54.79% and fourth component eigenvalues 1.209, cumulative 60.89% of the total variance. HCA was accomplished to classify the data according to the botanical origin and mineral content and to point out the similarity among different groups. The level of similarity in which explanations are joined together may be used to create a dendrogram. In the present work, the optimal linkage distance level was 410, represented by the dashed line in Figure 2. It represents a relative measure of similarity among analyzed honey samples. From Figure 2, it can be seen that three groups were formed, which can be identified as follows: eleven samples were clustered in group I (S1, S6, S8, S10, S14, S15, S21, S22, S23, S25, and S27); one sample in group II (S34); and twenty-five samples in group III (S2, S3, S4, S5, S7, S9, S11, S12, S13, S16, S17, S18, S19, S20, S24, S26, S28, S29, S30, S31, S32, S33, S35, S36, and S37). It can be observed that group I contains lavender, mild white mustard, milk thistle, carob tree, *Euphorbia*, and *Eucalyptus* honey types with high mineral contents, which showed similarities to each other. Group II contains sage honey type. Furthermore, group III comprises rosemary, thyme, orange tree, camphor, harmal, jujube tree, and many multifloral honey types with low levels of minerals, which correlated to each other. These results show that west Algerian honey samples vary according to their geographic origins and also to their mono or multifloral characteristics (Table 1). In addition, it is to underline that clear separation among the samples was obtained from several different geographic locations in western Algeria. From the PCA, it can be verified that the characteristics that influenced honey sample clustering the most were major elements K, Na, Ca, Mg, and Fe, whereas minor ones such as Cr, Co, V, W, As, Cd, and Pb were considered less important. In summary, the visualization of the data by HCA and PCA offers enough information to develop a classification method to determine the botanical origin of honey samples considering the mineral element composition.

## 4. Conclusion

The present chemical study provided a detailed picture of the trace element and heavy metal contents of natural honey from west Algeria, through the analysis of 19 elements in 37 types of unifloral honey (i.e., lavender, rosemary, mild white mustard, thyme, milk thistle, carob tree, orange tree, *Euphorbia*, *Eucalyptus*, camphor, jujube tree, sage, and harmal) and multifloral honey. Trace elements and heavy metals were determined and measured by ICP-MS and AAS methods. In summary, K was the most abundant element. K, Ca, Mg, and Na accounted for 97.92% of the total minerals present in honey investigated; this content places them among very dark and amber honey, which corresponds to their intrinsic golden/dark amber color. *Eucalyptus* honey contained the highest levels of K and Na. Sage honey is rich in Ca while milk thistle honey is rich in Mg. Additionally, lavender honey contained high Fe values while the carob tree is the richest in Zn. Mild white mustard honey contained highest level of Mn, whereas jujube tree and carob tree honey comprised Cu in high concentrations. Potentially toxic heavy metals and metalloids such as Pb, V, Cr, W, As, Co, and Cd were detected at concentrations <1 mg/kg. Furthermore, Ru, Rh, Pt, and Au were absent in all honey samples. The use of multivariate statistical methods such as HCA and PCA shows that mineral contents may represent a major discriminating strength based on the botanical origin of honey samples. Overall, our results indicated that the west Algerian honey is rich in essential minerals beneficial for human health. The levels of the trace elements and heavy metals were below the PTWI established by JECFA. Finally, it can be concluded that the west Algerian honey is uncontaminated and therefore represents a good indicator for monitoring environmental pollution with metals. Moreover, the levels of mineral contents are at safe levels for human consumption.

## Figures and Tables

**Table 1:** Geographical origins of honey samples from western regions of Algeria.

Region	Sample	Floral type	Scientific name	Family	Location	Season/year of harvest
Tlemcen						
	S1	Lavender	*Lavandula vera* D.C.	*Lamiaceae*	Sidi Djillali	Summer 2018
	S2	Rosemary	*Rosmarinus officinalis* L.	*Lamiaceae*	Sidi Djillali	Spring 2018
	S3	Multifloral	Multifloral	—	Sidi Djillali	Spring 2018
	S4	Multifloral	Multifloral	—	Sidi Djillali	Summer 2017
	S5	Multifloral	Multifloral	—	El Aricha	Summer 2017
	S6	Mild white mustard	*Sinapis alba* L.	*Brassicaceae*	Aïn Fezza	Summer 2017
	S7	Thyme	*Thymus vulgaris* L.	*Lamiaceae*	Beni Snous	Spring 2018
	S8	Milk thistle	*Silybum marianum* (L.) Gaertn.	*Asteraceae*	Beni Snous	Summer 2018
	S9	Multifloral	Multifloral	—	Oued Chouly	Autumn 2017
	S10	Carob tree	*Ceratonia siliqua* L.	*Fabaceae*	Oued Chouly	Autumn 2017
	S11	Thyme	*Thymus vulgaris* L.	*Lamiaceae*	Beni Mester	Spring 2017
	S12	Carob tree	*Ceratonia siliqua* L.	*Fabaceae*	Beni Ghazli	Spring 2017
	S13	Multifloral	Multifloral	—	Oued es Safsâf	Summer 2018
	S14	Multifloral	Multifloral	—	Sebaa Chioukh	Spring 2017
	S15	Multifloral	Multifloral	—	Hennaya	Summer 2017
	S16	Orange tree	*Citrus sinensis* L.	*Rutaceae*	Remchi	Spring 2017
	S17	Multifloral	Multifloral	—	Honaïne	Spring 2018
	S18	Milk thistle	*Silybum marianum* (L.) Gaertn.	*Asteraceae*	Honaïne	Summer 2018

Ain Temouchent						
	S19	Multifloral	Multifloral	—	Oulhaça El Gherarba	Spring 2018
	S20	Multifloral	Multifloral	—	Beni Ghanem	Summer 2018
	S21	Multifloral	Multifloral	—	Bouzedjar	Spring 2018

Sidi Bel Abbes						
	S22	*Euphorbia*	*Euphorbia* L.	*Euphorbiaceae*	Ras El Ma	Spring 2017
	S23	Milk thistle	*Silybum marianum* (L.) Gaertn.	*Asteraceae*	Telagh	Spring 2017
	S24	Multifloral	Multifloral	—	Lamtâr	Spring 2017
	S25	*Eucalyptus*	*Eucalyptus globulus* Labill.	*Myrtaceae*	Sidi Brahim	Spring 2017

Mostaganem						
	S26	Camphor	*Cinnamomum camphora* L.	*Lauraceae*	Sidi Ali	Autumn 2017
	S27	*Eucalyptus*	*Eucalyptus globulus* Labill.	*Myrtaceae*	Mostaganem	Summer 2017
	S28	Orange tree	*Citrus sinensis* L.	*Rutaceae*	Bouguirat	Spring 2017

Mascara						
	S29	Rosemary	*Rosmarinus officinalis* L.	*Lamiaceae*	Djebel Stamboul	Spring 2017

Tiaret						
	S30	Multifloral	Multifloral	—	Tiaret	Spring 2018

Naâma						
	S31	Multifloral	Multifloral	—	Aïn Sefra	Spring 2017
	S32	Jujube tree	*Ziziphus lotus* L.	*Rhamnaceae*	Aïn Sefra	Spring 2017
	S33	Jujube tree	*Ziziphus lotus* L.	*Rhamnaceae*	Aïn Ben Khelil	Spring 2017
	S34	Sage	*Salvia officinalis* L.	*Lamiaceae*	Naâma	Spring 2017
	S35	Harmal	*Peganum harmala* L.	*Zygophyllaceae*	Mecheria	Spring 2017

Bechar						
	S36	Multifloral	Multifloral	—	Djebel Antar	Winter 2017
	S37	Mild white mustard	*Sinapis alba* L.	*Brassicaceae*	Oued Zouzfana	Spring 2017

**Table 2:** Operating ICP-MS conditions.

Radio frequency power (W)	1370
Plasma gas flow rate (L/min)	18
Auxiliary gas flow rate (L/min)	1.70
Spray chamber T (°C)	2
Nebulizer gas flow (L/min)	1
Number of replicates	3

**Table 3:** Operating AAS conditions.

Elements	Na	Mg	K	Ca	Mn	Fe	Cu	Zn
Fuel flow (L/min)	2	2	2	2	2	2	2	2
Lamp current (mA)	—	4	—	10	5	5	4	5
Wavelength (nm)	589.0	202.6	766.5	422.7	279.5	248.3	324.8	213.9
Slit width (nm)	0.2	1.0	0.2	0.5	0.2	0.2	0.5	1.0
Air flow (L/min)	10	10	10	10	10	10	10	10

**Table 4:** Validation parameters of the analytical procedure for determination of minerals.

Element	Certified reference material analysis		Validation parameters			
	The result declared by the manufacturer	The result obtained in own research	LOD	LOQ	Recovery (%)	Uncertainty (%)
As (*μ*g/kg)	112.6^a^	112.21	0.5	1.0	100.35	16
	39^b^	42.10			92.64	21
Au (*μ*g/kg)	—	—	2.3	4.6	109.12	26
Ca (mg/kg)	50450^a^	53700.23	12.1	24.2	93.95	17
	2500^b^	2356.45			106.09	19
Cd (*μ*g/kg)	1517^a^	1498.03	0.1	0.2	101.27	13
	233^b^	276.20			84.36	16
Co (*μ*g/kg)	577.3^a^	774.82	0.1	0.2	74.51	18
	61^b^	62.76			97.20	20
Cr (*μ*g/kg)	1988^a^	2123.20	8.0	16.0	93.63	11
	61^b^	62.76			97.20	14
Cu (mg/kg)	4.7^a^	4.92	0.7	1.4	95.68	19
	2.8^b^	3.1			90.32	25
Fe (mg/kg)	367.5^a^	391.09	3.7	7.2	93.97	10
	46^b^	39.21			117.32	15
K (mg/kg)	26760^a^	30207.00	23.2	46.4	88.59	15
	4170^b^	4376.00			95.29	14
Mg (mg/kg)	12000^a^	12096.00	9.3	18.6	99.21	17
	2610^c^	2734.00			95.46	12
Mn (mg/kg)	246.3^a^	242.02	0.5	1.0	101.77	19
	488^b^	460.50			105.97	11
Na (mg/kg)	136.1^a^	157.15	6.0	12.0	86.61	14
	63^b^	67.89			92.80	20
Pb (*μ*g/kg)	167^b^	163.58	0.2	0.4	102.09	23
Pt (*μ*g/kg)	—	—	3.0	6.0	107.12	24
Ru (*μ*g/kg)	—	—	2.5	5.0	85.34	33
Rh (*μ*g/kg)	—	—	2.2	4.4	105.23	28
V (*μ*g/kg)	835^a^	867.58	0.4	0.8	96.24	25
W (*μ*g/kg)	—	—	4.1	8.2	88.12	35
Zn (mg/kg)	30.94^a^	36.49	0.1	0.2	84.79	21
	38^b^	38.50			98.70	16

“a” NIST-1573a Tomato Leaves Standard Reference Materials; “b” NIST-1575a Pine Needles (Pinus taeda) Standard Reference Materials; “c” Certified single-element standard solutions.

**Table 5:** Distribution data for Cr, Co, V, W, Ru, Rh, Pt, Au, As, Cd, and Pb (*μ*g/kg) in west Algerian honey.

Sample	Cr^b,c^	Co^b,c^	V^b,c^	W^b,c^	Ru^b,c^	Rh^b,c^	Pt^b,c^	Au^b,c^	As^b^	Cd^b,c^	Pb^b,c^
S1	70.11	9.01	8.62	—^d^	—^d^	—^d^	—^d^	—^d^	—^d^	1.27	21.44
S2	55.56	8.73	9.00	—^d^	—^d^	—^d^	—^d^	—^d^	4.93	0.83	16.62
S3	38.73	10.33	4.83	—^d^	—^d^	—^d^	—^d^	—^d^	7.70	1.09	9.73
S4	37.20	12.14	10.04	—^d^	—^d^	—^d^	—^d^	—^d^	21.56	0.36	0.54
S5	34.07	20.80	6.88	—^d^	—^d^	—^d^	—^d^	—^d^	3.38	0.90	20.25
S6	56.21	4.57	7.30	—^d^	—^d^	—^d^	—^d^	—^d^	4.70	0.79	21.05
S7	27.63	5.96	1.57	—^d^	—^d^	—^d^	—^d^	—^d^	1.75	8.14	9.87
S8	45.89	4.73	4.94	—^d^	—^d^	—^d^	—^d^	—^d^	18.20	0.24	—^d^
S9	41.02	17.32	6.00	—^d^	—^d^	—^d^	—^d^	—^d^	4.05	0.54	11.94
S10	87.37	12.67	7.29	—^d^	—^d^	—^d^	—^d^	—^d^	5.36	0.55	14.83
S11	43.74	6.61	79.73	—^d^	—^d^	—^d^	—^d^	—^d^	3.95	1.50	23.20
S12	53.39	1.45	4.48	—^d^	—^d^	—^d^	—^d^	—^d^	5.54	0.77	10.78
S13	59.05	3.36	5.12	—^d^	—^d^	—^d^	—^d^	—^d^	4.80	0.97	9.21
S14	74.83	4.20	13.98	—^d^	—^d^	—^d^	—^d^	—^d^	6.43	1.04	13.75
S15	29.60	3.56	6.52	—^d^	—^d^	—^d^	—^d^	—^d^	5.27	1.26	17.93
S16	38.58	1.02	1.44	—^d^	—^d^	—^d^	—^d^	—^d^	1.61	1.05	12.84
S17	39.84	0.36	2.10	—^d^	—^d^	—^d^	—^d^	—^d^	1.68	—^d^	132.73
S18	41.39	3.29	3.42	—^d^	—^d^	—^d^	—^d^	—^d^	6.28	1.14	15.28
S19	55.46	5.77	13.84	—^d^	—^d^	—^d^	—^d^	—^d^	6.31	0.84	14.39
S20	34.15	2.82	3.23	—^d^	—^d^	—^d^	—^d^	—^d^	5.93	0.83	10.18
S21	42.62	4.60	10.26	10.65	—^d^	—^d^	—^d^	—^d^	6.68	3.38	89.44
S22	39.17	8.42	4.58	—^d^	—^d^	—^d^	—^d^	—^d^	6.23	1.43	14.23
S23	46.89	5.25	9.09	17.18	—^d^	—^d^	—^d^	—^d^	7.73	0.41	40.55
S24	30.18	3.25	6.40	—^d^	—^d^	—^d^	—^d^	—^d^	6.28	0.98	14.95
S25	29.13	4.18	4.55	11.10	—^d^	—^d^	—^d^	—^d^	8.19	0.86	24.57
S26	58.86	7.82	106.51	—^d^	—^d^	—^d^	—^d^	—^d^	3.95	0.69	9.74
S27	27.36	3.07	4.75	—^d^	—^d^	—^d^	—^d^	—^d^	4.92	0.90	26.52
S28	37.06	7.04	7.68	—^d^	—^d^	—^d^	—^d^	—^d^	7.38	0.56	11.15
S29	27.81	1.89	0.81	—^d^	—^d^	—^d^	—^d^	—^d^	2.70	1.05	11.91
S30	37.61	16.64	7.65	—^d^	—^d^	—^d^	—^d^	—^d^	2.81	2.94	91.36
S31	62.04	17.78	27.11	—^d^	—^d^	—^d^	—^d^	—^d^	10.41	1.14	24.70
S32	53.73	16.95	15.35	—^d^	—^d^	—^d^	—^d^	—^d^	5.64	0.89	21.32
S33	50.33	12.11	11.62	—^d^	—^d^	—^d^	—^d^	—^d^	2.90	0.99	20.95
S34	44.25	12.94	10.24	—^d^	—^d^	—^d^	—^d^	—^d^	4.35	1.00	74.99
S35	72.11	21.19	16.47	24.30	—^d^	—^d^	—^d^	—^d^	8.30	0.84	16.41
S36	55.96	1.01	44.33	—^d^	—^d^	—^d^	—^d^	—^d^	4.95	1.02	8.21
S37	47.23	6.74	12.37	—^d^	—^d^	—^d^	—^d^	—^d^	3.96	1.19	27.30

Statistics											
Mean	46.65	7.83	13.52	15.81	—^d^	—^d^	—^d^	—^d^	5.86	1.23	24.73
Max.	87.37	21.19	106.51	24.30	—^d^	—^d^	—^d^	—^d^	21.56	8.14	132.73
Min.	27.36	0.36	0.81	10.65	—^d^	—^d^	—^d^	—^d^	1.61	0.24	0.54
SD	14.43	5.82	21.05	4.93	—^d^	—^d^	—^d^	—^d^	4.03	1.32	27.59

^b,c^trace elements/heavy metals; ^d^concentrations were lower than LOQ; SD: standard deviation.

**Table 6:** Distribution data for K, Na, Ca, Mg, Cu, Mn, Fe, and Zn (mg/kg) in west Algerian honey.

Sample	K^a^	Na^a^	Ca^a^	Mg^a^	Cu^b,c^	Mn^b,c^	Fe^b,c^	Zn^b,c^	Total minerals
S1	808.00	21.60	56.20	142.00	3.66	13.30	59.60	2.62	1107.58
S2	460.00	49.20	58.50	126.00	5.70	10.80	24.50	2.39	737.53
S3	418.00	37.00	64.90	142.00	4.46	11.10	24.40	4.41	706.68
S4	451.00	30.70	73.60	92.70	9.62	7.67	26.10	3.38	695.19
S5	327.00	34.10	50.60	77.60	—^d^	5.52	19.60	0.22	514.92
S6	946.00	146.00	104.00	149.00	5.92	13.90	23.20	3.39	1392.47
S7	241.00	13.30	33.10	159.00	5.08	12.70	22.60	5.29	492.53
S8	790.00	22.10	97.80	162.00	4.81	10.50	11.70	4.42	1104.26
S9	441.00	97.80	85.80	88.80	—^d^	7.85	34.60	9.33	765.57
S10	667.00	64.40	205.00	120.00	7.86	10.80	57.30	13.90	1147.04
S11	589.00	104.00	102.00	79.00	3.44	7.61	33.70	4.98	924.28
S12	153.00	15.30	33.50	116.00	2.80	11.50	24.30	3.08	359.77
S13	572.00	102.00	104.00	127.00	1.66	13.70	16.30	4.99	942.24
S14	989.00	77.10	377.00	137.00	—^d^	11.50	17.60	3.45	1613.00
S15	722.00	32.30	99.90	69.10	5.29	1.36	26.80	0.62	957.88
S16	254.00	62.30	81.10	89.20	1.95	9.26	26.00	4.37	528.41
S17	375.00	129.00	33.30	134.00	7.16	12.10	14.90	4.59	710.58
S18	526.00	68.20	53.80	133.00	—^d^	13.40	21.60	5.20	821.65
S19	400.00	110.00	119.00	118.00	2.13	8.12	22.60	2.33	782.65
S20	247.00	26.50	70.00	112.00	—^d^	10.20	25.10	2.29	493.45
S21	776.00	23.60	80.80	51.60	6.08	3.83	24.00	2.59	969.00
S22	675.00	281.00	100.00	99.50	6.61	7.38	18.10	3.30	1191.51
S23	802.00	71.70	215.00	55.30	4.67	1.47	26.20	3.63	1180.73
S24	425.00	156.00	101.00	20.80	—^d^	1.93	8.48	1.87	715.40
S25	777.00	275.00	287.00	77.80	—^d^	1.69	11.30	2.82	1433.12
S26	470.00	92.50	132.00	57.00	4.17	9.25	21.10	3.41	789.77
S27	987.00	23.60	165.00	124.00	7.25	6.55	21.80	4.75	1340.80
S28	338.00	179.00	91.00	66.80	—^d^	6.50	19.10	2.46	703.31
S29	384.00	102.00	77.70	85.40	5.46	8.63	26.30	3.17	692.92
S30	471.00	44.30	70.70	78.40	4.88	3.45	24.10	1.44	699.04
S31	504.00	64.60	90.80	80.20	3.75	6.10	35.80	2.75	788.61
S32	442.00	165.00	117.00	92.80	3.59	4.70	38.30	3.62	867.55
S33	510.00	51.20	70.70	123.00	8.97	6.15	29.50	1.78	801.68
S34	441.00	114.00	502.00	111.00	—^d^	3.90	25.60	3.68	1201.59
S35	460.00	66.10	71.90	98.60	2.52	7.28	29.60	2.69	739.34
S36	411.00	35.90	49.00	42.50	—^d^	6.46	28.00	2.58	576.28
S37	452.00	97.40	90.50	92.60	2.78	5.87	15.40	1.93	758.88
Statistics									
Mean	532.46	83.40	113.92	100.83	4.90	7.95	25.28	3.61	871.55
Max.	989.00	281.00	502.00	162.00	9.62	13.90	59.60	13.90	1613.00
Min.	153.00	13.30	33.10	20.80	1.66	1.36	8.48	0.22	359.77
SD	212.69	64.85	95.38	33.88	1.65	3.66	10.39	2.35	290.66

^a^Major elements; ^b^trace elements; ^b,c^trace elements/heavy metals; ^d^concentrations were lower than LOQ; SD: standard deviation.

**Table 7:** A comparison among reports regarding mineral content in honey samples from Mediterranean regions.

Element	Present study	Morocco [38]	Morocco [55]	Tunisia [39]	Portugal [43]	Portugal [44]	Spain [46]	Spain [47]	Spain [48]	Spain [49]	Spain [50]
		*N* = 29	*N* = 8	*N* = 6	*N* = 38	*N* = 16	*N* = 40	*N* = 8	*N* = 60	*N* = 140	*N* = 41
Cr	27.36–87.37 *μ*g/kg	—	—	0.02–0.32	—	—	—	—	0.049–4.480	—	—
Co	0.36–21.19 *μ*g/kg	—	nd	—	—	—	—	—	0.015–0.720	—	—
V	0.81–106.51 *μ*g/kg	—	—	—	—	—	—	—	—	—	—
W	10.65–24.30 *μ*g/kg	—	—	—	—	—	—	—	—	—	—
Ru	˂LOQ	—	—	—	—	—	—	—	—	—	—
Rh	˂LOQ	—	—	—	—	—	—	—	—	—	—
Pt	˂LOQ	—	—	—	—	—	—	—	—	—	—
Au	˂LOQ	—	—	—	—	—	—	—	—	—	—
As	1.61–21.56 *μ*g/kg	—	—	—	—	—	—	—	—	—	—
Cd	0.24–8.14 *μ*g/kg	—	nd	—	—	—	—	—	nd–0.355	4.21–4.56 *μ*g/kg	—
Pb	0.54–132.73 *μ*g/kg	≤0.1	nd	0.01–0.05	—	—	—	—	—	46.32–31.50 *μ*g/kg	—
K	153.00–989.00	256–1023	644.02–1883.15	172.48–976.75	117.55–2590.60	42.98–1352.90	434.1–1935	545.45–5570.73	514–6785	—	1615–3770
Na	13.30–281.00	18.81–118.74	367.52–855.24	251.34–521.22	90.22–727.79	0.36–95.13	11.69–218.5	9.18–151.65	38–476	—	11–84
Ca	33.10–502.00	19.71–200.1	129.35–688.43	113.85–221.07	6.24–134.35	2.77–234.63	42.59–341.0	15.14–181.69	107–420	—	68–476
Mg	20.80–162.00	19.85–45.84	18.42–131.21	—	10.62–70.41	1.79–230.81	13.26–74.38	42.11–1078.95	19–173	—	30–402
Cu	1.66–9.62	≤0.1	0.11–1.82	0.12–0.34	—	0.00–5.35	0.531–2.117	nd	0.547–2.300	0.74–1.88	1–7
Mn	1.36–13.90	0.28–1.74	—	—	—	0.00–22.62	0.133–9.471	—	3.4–45	—	—
Fe	8.48–59.60	1.46–13.95	0.71–4.68	0.83–3.54	—	0.18–2.68	—	nd–7.07	0.71–60	2.26–4.70	0–7
Zn	0.22–13.90	≤0.1–0.69	1.41–4.26	0.42–2.06	—	0.03–3.29	1.332–7.825	0.30–7.06	0.93–5.9	2.34–3.47	0–7

From each report, the min-max range is mentioned. All values are in mg/kg, unless stated otherwise. nd: not detected; ˂LOQ: concentrations were lower than LOQ; *N*: the number of honey samples.

**Table 8:** A comparison among reports regarding mineral content in honey samples from Mediterranean regions.

Element	Spain [51]	France [53]	France [54]	Italy [24]	Italy [22]	Turkey [19]	Greece [25]	Cyprus [25]	Egypt [25]	Palestine [40]	Palestine [41]	Syria [42]	Libya [45]
	*N* = 25	*N* = 86	*N* = 40	*N* = 40	*N* = 51	*N* = 71	*N* = 12	*N* = 14	*N* = 8	*N* = 21	*N* = 10	*N* = 6	*N* = 8
Cr	—	0.08–0.36	—	10–328	—	<1 *μ*g/kg	<0.12	<0.12	<0.12	0.00–0.51	—	<0.018–0.054	—
Co	—	0.10–0.23	—	1.0–17	2.9–30.2	—	<0.03	<0.03	<0.03	—	—	—	—
V	—	—	—	<3–24	—	—	<0.11	<0.11	<0.11	—	—	—	—
W	—	—	—	—	—	—	—	—	—	—	—	—	—
Ru	—	—	—	—	—	—	—	—	—	—	—	—	—
Rh	—	—	—	—	—	—	—	—	—	—	—	—	—
Pt	—	—	—	—	—	—	—	—	—	—	—	—	—
Au	—	—	—	—	—	—	—	—	—	—	—	—	—
As	—	—	nd–8.0	<25	2.8–11.1	—	<0.08	<0.08	<0.08	—	—	—	0.006–0.018
Cd	—	0.08–0.25	1–22	1.3–4.2	1.0–15.3	<1 *μ*g/kg	<0.05	<0.05	<0.05	—	—	—	0.125–0.150
Pb	—	0.28–1.08	3–101	9–209	28–304	<1 *μ*g/kg	<0.08	<0.08	<0.08	—	0.51–0.94	<0.082	2.42–10.98
K	261–1380	—	—	237–6520	178–4140	1.18–268	—	—	—	42.80–585.00	203.92–495.48	38.2–174	1120.1–1980.6
Na	256–501	—	—	4.8–176	60–147	0.48–13.1	—	—	—	41.80–306.30	35.19–196.51	—	506.8–804.6
Ca	110–248	8.90–130.90	36–219	<43–283	159–373	<0.001–4.5	15.22–65.93	23.66–143.47	44.79–112.10	44.50–150.70	64.49–138.41	43.3–118	923.92–1117.5
Mg	37–139	3.62–68.78	33–145	6.2–148	22–159	—	20.13–109.53	9.24–80.48	12.17–22.04	12.30–46.70	20.48–54.15	—	—
Cu	—	0.06–1.71	—	0.06–5.4	0.20–2.79	<1–929 *μ*g/kg	<0.35	<0.35	<0.35	0.00–1.52	0.61–1.22	0.616–2.21	—
Mn	—	0.11–42.81	1.4–12.8	0.09–2.8	0.13–16.9	<1–274 *μ*g/kg	0.26–5.54	0.07–4.11	0.17–0.55	0.11–0.99	—	0.153–1.69	—
Fe	—	0.56–86.76	—	<1–4.4	1.0–6.3	<1–7254.62 *μ*g/kg	0.75–4.16	0.96–3.97	2.18–8.45	2.00–10.80	2.25–8.75	1.46–16.9	—
Zn	—	0.17–6.42	nd–1.4	<0.5–8.9	0.72–3.66	<1–237 *μ*g/kg	0.97–9.30	0.86–6.94	0.55–1.68	1.00–19.90	0.13–25.20	0.206–2.76	—

From each report, the min-max range is mentioned. All values are in mg/kg, unless stated otherwise. nd: not detected; ˂ LOQ: concentrations were lower than LOQ; *N*: the number of honey samples.

**Table 9:** Variance estimates (eigenvalues) and cumulative percentage of total variance (%) obtained by PCA considering 37 honey samples and trace elements and heavy metals.

Principal components	Honey samples	
	Eigenvalues	Cumulative %
1	1.937	30.38
2	1.607	54.33
3	1.562	54.79
4	1.209	60.89

## Data Availability

The data used to support the findings of this study are included within the article.
